# COVID-19 and Diabetes

**DOI:** 10.3390/jcm10225341

**Published:** 2021-11-16

**Authors:** Virginia Bellido, Antonio Pérez

**Affiliations:** 1 Endocrinology and Nutrition Department, Hospital Universitario Virgen del Rocío, 41013 Sevilla, Spain; virginiabellido@gmail.com; 2 Endocrinology and Nutrition Department, Hospital de la Santa Creu i Sant Pau, Institut d’Investigació Bio-Mèdica Sant Pau, 08041 Barcelona, Spain; 3 Centro de Investigación Biomédica en Red de Diabetes y Enfermedades Metabólicas Asociadas (CIBERDEM), Universitat Autònoma de Barcelona, 08193 Barcelona, Spain

**Keywords:** diabetes mellitus, COVID-19, hyperglycemia, glycemic control, blood glucose monitoring, telemedicine

## Abstract

Diabetes mellitus (DM) is one of the most common comorbid conditions in persons with COVID-19 and a risk factor for poor prognosis. The reasons why COVID-19 is more severe in persons with DM are currently unknown although the scarce data available on patients with DM hospitalized because of COVID-19 show that glycemic control is inadequate. The fact that patients with COVID-19 are usually cared for by health professionals with limited experience in the management of diabetes and the need to prevent exposure to the virus may also be obstacles to glycemic control in patients with COVID-19. Effective clinical care should consider various aspects, including screening for the disease in at-risk persons, education, and monitoring of control and complications. We examine the effect of COVID-19 on DM in terms of glycemic control and the restrictions arising from the pandemic and assess management of diabetes and drug therapy in various scenarios, taking into account factors such as physical exercise, diet, blood glucose monitoring, and pharmacological treatment. Specific attention is given to patients who have been admitted to hospital and critically ill patients. Finally, we consider the role of telemedicine in the management of DM patients with COVID-19 during the pandemic and in the future.

## 1. Diabetes and COVID-19

Diabetes mellitus (DM) is a medical condition that can have a considerable impact on affected persons and on society owing to the high costs associated with its care, especially those arising from complications. The situation becomes more serious during a pandemic, such as that of COVID-19, as having DM entails a greater risk of extended hospital stay and death. In addition to the direct effects on health, the absence of regular care owing to closure of outpatient clinics and social isolation—combined with changes in diet, physical activity, and personal care—favors deterioration of disease control and hampers detection of complications. All of the above factors could prove responsible for poorer clinical outcomes in patients with DM

This study examines the impact of the COVID-19 pandemic on persons with DM and covers implications for health in the short and long terms. It also examines how to address the threat to persons with DM arising from more limited health services and changes in lifestyle resulting from the pandemic.

## 2. COVID-19 in Persons with Diabetes

### 2.1. Impact of Diabetes on COVID-19

DM is one of the most common comorbid conditions in persons with COVID-19, and while the presence of DM does not seem to increase the risk of infection [1], it is a risk factor for poor prognosis [2,3]. The prevalence of DM in persons with COVID-19 varies widely according to published series, from 7% to 30% [4]. A meta-analysis by Fadini et al. [1] in Italy examined 12 studies performed in China and, including outpatients and hospitalized patients, found the prevalence of DM to be 10.3%, which overlapped with or was even slightly inferior to the prevalence of DM in the Chinese population adjusted for age. In the whole-population study by Barron et al. [5] in England, which included 61,414,470 live individuals registered in primary care, 0.4% had been diagnosed with type 1 diabetes (T1D), 4.7% had a diagnosis of type 2 diabetes (T2D), and 0.1% had other types of DM. The odds ratios (ORs) for in-hospital COVID-19-related death were 3.51 (95% CI, 3.16–3.90) in people with type 1 diabetes and 2.03 (1.97–2.09) in people with type 2 diabetes after adjustment for age, sex, deprivation, ethnicity, and geographical region. These effects were attenuated, reaching ORs of 2.86 (2.58–3.18) for type 1 diabetes and 1.80 (1.75–1.86) for type 2 diabetes when also adjusted for previous hospital admissions with coronary heart disease, cerebrovascular disease, and heart failure. Various studies have shown that DM is present in approximately 20% of persons infected by type 2 coronavirus causing severe acute respiratory syndrome (SARS-CoV-2) and that it is one of the most common comorbid conditions together with arterial hypertension, obesity, and cardiovascular disease [6,7,8].

Once COVID-19 is acquired, DM might increase the severity and mortality of the disease to the extent that patients with uncontrolled hyperglycemia or DM have a greater risk of respiratory failure and cardiac complications and more than double the probability of being admitted to the intensive care unit (ICU). Moreover, mortality is 3-fold greater than in patients without DM or uncontrolled hyperglycemia [2,3,5,9,10]. In the study by Barron et al. [5], 30% of deaths from COVID-19 were in persons with DM, and the risk of death was almost 3-fold greater for persons with T1D and almost double for those with T2D than in those who did not have DM. In addition to the impact on health, the COVID-19 pandemic considerably affects the use of health care resources and costs. In the USA, the average direct medical cost during the course of the infection was estimated to double or triple in patients with comorbid conditions, such as DM [11].

The reasons why COVID-19 is more severe in persons with DM are currently unknown [12]. Potential pathophysiological mechanisms that contribute to the increase in morbidity and mortality include presence of an underlying chronic inflammatory state in DM, impaired immune response, and coagulation abnormalities. The high prevalence of DM in severe cases of COVID-19 could reflect the greater prevalence of T2D in elderly people. Furthermore, DM in the elderly is associated with cardiovascular diseases and obesity, which in themselves go some way to explaining the association with the fatal outcome of COVID-19. However, the association between DM and a poorer prognosis is maintained in non-hypertensive younger patients [9]. Current studies have shown that hyperglycemia at admission to hospital is a predictor of death and other severe outcomes of COVID-19 [13,14]. The Spanish registry of the Spanish Society of Internal Medicine for COVID-19 [14], which included 11,312 patients (18.9% with previous DM) hospitalized with COVID-19 in 109 hospitals, showed that patients who were not critically ill but presented with hyperglycemia at admission, irrespective of whether they had previously had DM or not, were more likely to develop complications and die and that this risk increased with the grade of hyperglycemia (blood glucose >180 mg/dL (>10 mmol/L); HR for mortality, 1.50; 95% CI, 1.31–1.73; and blood glucose 140–180 mg/dL (7.8–10 mmol/L); HR, 1.48; 95% CI, 1.29–1.70). A retrospective study of 1544 patients with COVID-19 from 91 hospitals in the USA [13] showed that both hyperglycemia and hypoglycemia were associated with poor outcomes in patients with COVID-19. In addition, although clearly insufficient, available data support the fact that optimal control of glycemia during hospital stay could prove to be beneficial in terms of clinical outcomes in patients with DM and COVID-19. A study of 59 patients with COVID-19 admitted to two Italian hospitals [15] showed that treatment with insulin infusion until blood glucose levels of <140 mg/dL (<7.8 mmol/L) were reached in 15 subjects with hyperglycemia and improved their prognosis with respect to patients who had not received an insulin infusion. Furthermore, levels of interleukin 6 and D-dimer decreased once hyperglycemia was treated.

Specific mechanisms with a potential role in COVID-19 infection include angiotensin II–converting enzyme (ACE2) and dipeptidyl peptidase 4 (DPP4) [12,16]. ACE2 has been identified as a coronavirus surface protein receptor. COVID-19 reduces expression of ACE2, thus inducing cell damage, hyperinflammation, and respiratory failure. Acute hyperglycemia upregulates expression of ACE2 in cells, thus potentially facilitating the entry of viral cells [3,17]. However, we know that chronic hyperglycemia negatively regulates expression of ACE2, leaving cells vulnerable to the effects of the virus [18]. Cell studies have identified DPP4 as a functional receptor for human coronavirus–Erasmus Medical Center (HCoV-EMC) and antibodies targeting DPP4 inhibited infection of primary cells by HCoV-EMC [16]. At present, it is unknown whether these mechanisms can also be applied to COVID-19 and whether treatment of DM with DPP4 inhibitors in clinical practice affects the course of the infection.

### 2.2. Impact of COVID-19 on Diabetes

DM is not only a risk factor for greater severity of COVID-19; in fact, the disease affects persons with DM directly, as in other viral infections (by worsening previous DM and even inducing new-onset DM), or indirectly, as a consequence of the restrictions arising from lockdown during the COVID-19 pandemic.

#### 2.2.1. Effects of COVID-19 on Glycemic Control

The scarce data available on patients with DM hospitalized because of COVID-19 show that glycemic control is inadequate [19,20]. A study that analyzed glycemic outcomes during admission found that 39.1% of values were over 180 mg/dL (10 mmol/L) and that the mean blood glucose level was over 180 mg/dL (10 mmol/L) for 37.8% of hospital stay [19]. In a study carried out in the USA [13], more than half of patients admitted to the ICU (56%) and outside the ICU (53%) did not reach their target blood glucose levels during the first two or three days; a study performed in China found that 56.6% of capillary blood glucose test results were higher than the recommended target (140–180 mg/dL) (7.8–10 mmol/L) [20]. In patients who required insulin, SARS-CoV-2 infection was associated with very high insulin requirements, reaching doses of 201 IU/d (2.2 IU/kg/d) [21]; these high values are associated with levels of inflammatory cytokines. Decompensation in the form of diabetic ketoacidosis has been reported in patients with T2D and COVID-19, as is the case in other severe infections. One systematic review reported that 77% of patients with COVID-19 who developed ketoacidosis had underlying T2D, and DM was diagnosed in 10 patients at admission; of these, seven had glycated hemoglobin >9.5% [22]. The pathophysiology of these manifestations of DM is complex and probably goes beyond the well-established stress response associated with severe disease and the toxicity induced by persistently elevated glucose concentrations. The proinflammatory medium induced by COVID-19 can lead to a high degree of insulin resistance, thus increasing insulin requirements. Pancreatic β cells express ACE2, which can lead the virus to enter the pancreatic islets and damage the β cells, thus causing insulin deficiency. This effect worsens the course of DM and causes acute hyperglycemia, even in persons without DM [23]. Insulin deficiency in the setting of marked insulin resistance might also explain the common finding of cases of severe diabetic ketoacidosis and ketosis at admission [24]. Furthermore, drugs that are commonly used in clinical practice for patients with COVID-19, such as systemic corticosteroids and antiviral agents, worsen glycemic control and lead to marked glycemic excursions over a 24-h period [25,26]. In addition, there have been reports of a high number of hypoglycemic episodes at admission, probably favored by the anorexia induced by COVID-19 and without the concomitant adjustment of glucose-lowering drugs [20,27]. Finally, the fact that patients with COVID-19 are cared for by health professionals with limited experience in the management of hyperglycemia and the need to prevent exposure to the virus may also be obstacles to glycemic control in patients with COVID-19. Therefore, impaired glycemic control in patients with DM and hyperglycemia in patients without previous DM is considered a complication of COVID-19.

#### 2.2.2. Effects on Diabetes of the Restrictions Arising from the COVID-19 Pandemic

The COVID-19 pandemic was and continues to be a considerable challenge for people with DM since their normal routines have been interrupted in order to comply with social distancing measures. The immediate consequence is that a patient’s ability to gain access to and receive medical care, obtain medication and material for control of DM, and maintain a healthy lifestyle and social connections have been considerably affected. While information on the indirect consequences of the COVID-19 pandemic on DM is limited, we are now seeing data that make it possible to evaluate the impact of the first wave.

Studies in patients with T1D who use continuous glucose monitoring (CGM) or flash glucose monitoring (FGM) have shown that during lockdown, there was no deterioration in glycemic control or even beneficial effects [28,29]. A recent meta-analysis including 3441 individuals with T1D with CGM or FGM showed that, during the lockdown period, time in range 70–180 mg/dL increased by 3.05% (95% CI, 1.67–4.43%; *p* < 0.0001), while time above range (>180 mg/dL and > 250 mg/dL) declined by 3.39% (–5.14 to –1.63%) and 1.96% (−2.51 to −1.42%), respectively (*p* < 0.0001 for both) [30]. It has been speculated that this improvement could be associated with the ability to spend more time monitoring DM, having more regular timetables, and experiencing less stress associated with going to and from work. However, these findings may not be applicable to people with T1D who are less motivated to control their disease, who do not use CGM, or whose social-occupational situation competes for the time spent on managing DM. Of the 763 persons with T1D who participated in the Taking Control of Your Diabetes study in the USA, 46% reported that the pandemic hampered their management of DM. Furthermore, in approximately 25%, there was an increase in the frequency of high blood glucose levels and variations in blood glucose [31]. Finally, of the 603 patients with T1D who participated in a web survey in Spain, two-thirds reported impaired glycemic control, and 4 out of 10 reported weight gain [32].

The T2D population is much more heterogeneous than the T1D population in relevant aspects such as treatment, monitoring, and ability to self-adjust treatment and use remote consultation tools. The results of the survey among the subjects of Taking Control of Your Diabetes (763 persons with T1D and 619 with T2D) show that the impact of lockdown on management of DM was similar in both populations [31]. Patients were mainly non-Hispanic White, were educated to a high level, and had good control of their glycemia. Furthermore, in the case of patients with T2D, 46% received treatment with insulin, and 25% used CGM. A study of 114 patients with T2D followed at a tertiary center in Italy found that lockdown led to poorer metabolic control in the short term in 26% of patients who had previously been well controlled [33]. Given the characteristics of the populations studied and the care setting, these data are not applicable to the general population with T2D, especially in those patients who require care from the health system for monitoring of control and intensification of treatment. In addition, given that published data are very short term and that T2D is progressive, we might expect that the absence of or reduction in monitoring and in intensification of treatment leads to more frequently impaired control in the longer term. Data from the electronic medical records of a cohort of 13,352,550 patients from 1709 primary care centers in the United Kingdom followed between March and April 2020 raise particular concern over the considerable reduction (77–84%) in testing of glycated hemoglobin and in the prescription of metformin and insulin, especially in older persons with T2D [34]. Similar changes in glucose-lowering therapy were reported in patients with T2D in Germany between January and July 2019 (*N* = 79,268) and between January and July 2020 (*N* = 85,046). Compared with 2019, the number of persons with ≥1 change in medication fell in 2020, as follows: DPP4 inhibitors, –15%; sodium-glucose co-transporter 2 (SGLT2) inhibitors, –3%; glucagon-like peptide-1 (GLP1) receptor agonists, 0%; other oral hypoglycemic drugs, –6%; and insulin, –21% [35]. Another indirect consequence of the COVID-19 pandemic in T2D patients is the effect on diagnosis of DM, which requires specific diagnostic tests that must be performed in a clinical setting. The first four months of lockdown in the United Kingdom saw a reduction of 70% in new diagnoses of T2D; that is, more than 45,000 diagnoses were either not made or delayed during this period [34]. Taken together, these data are worrying since absence of or delaying diagnosis and monitoring of DM hampers decisions on therapy aimed at improving metabolic control and preventing the development or progression of potentially severe complications in the long term.

## 3. Treatment of Diabetes during the COVID-19 Pandemic: Disease Management and Drug Therapy in Various Scenarios

### 3.1. Diabetes Patients without COVID-19: Lockdown and Lack of Physical Exercise

People with DM must be aware of the importance of maintaining good glycemic control during the COVID-19 pandemic since stability of blood glucose levels can help to ensure a milder clinical course if the individual becomes infected [36]. Good glycemic control depends on tailoring therapy to the individual patient’s situation. A balanced diet, regular physical exercise, psychological stability, and adequately adjusted treatment are key elements when attempting to achieve objectives for disease control. It is also important to control comorbid conditions associated with DM, such as routine vaccination against pneumococcus and influenza (Table 1) [37]. It is as well a priority for people with diabetes to receive the COVID-19 vaccine. Clinical data have shown a robust neutralizing antibody response in patients with diabetes [38]. However, recent data have shown that hyperglycemia at the time of COVID-19 vaccination worsens the immune response, whereas achieving adequate glycemic control during the post-vaccination period improves the immune response [39]. Therefore, we need to focus on achieving good glycemic control, which can play a role in clinical COVID-19 outcomes and vaccine efficiency

Table 1 summarizes the main recommendations for the prevention of COVID-19 in people with diabetes.

General public health measures issued by health authorities (e.g., social distancing, hand washing, masks, and lockdown) should receive specific emphasis in people with DM. Similarly, telemedicine should be preferred to limit exposure of people with DM while at the same time guaranteeing continuity of care [44].

Greater vigilance is warranted for early detection of signs and symptoms—even atypical ones—of SARS-CoV-2 infection in patients with DM. A lower clinical threshold for suspicion of COVID-19 should be established in order to avoid delays in health care and adverse outcomes in this population [45].

### 3.2. Patients with Diabetes and COVID-19 Who Have Not Been Admitted to Hospital

Most persons with COVID-19 and DM develop mild disease that can be managed at home according to local guidelines. In these cases, regular contact with and follow-up by health services are crucial for identifying impaired control or clinical status. Similarly, optimization of glycemic control is key if we are to reduce the risk of severe disease. Therefore, blood glucose levels should be monitored frequently, and patients should follow a healthy diet, ensure appropriate fluid intake, and adjust treatment in cases of impaired glycemic control [37]. Integrated management of comorbid conditions and associated cardiovascular risk factors are equally important during this period [37].

**Figure 1:**
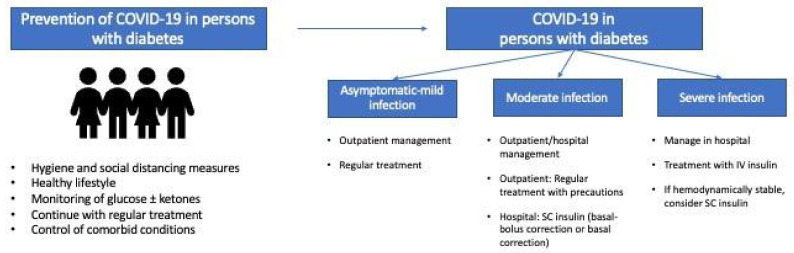
General recommendations for prevention and management of COVID-19 in persons with diabetes.

Figure 1 shows general recommendations for prevention and management of COVID-19 in people with diabetes.

#### 3.2.1. Objectives of Glycemic Control

The objectives of glycemic control should be tailored according to age, comorbid conditions, complications, and the clinical severity of infection. It is generally recommended to maintain preprandial glucose levels between 70 and 130 mg/dL and postprandial levels <180 mg/dL. In the case of elderly or frail patients, more easily achievable objectives can be set, with priority given to avoiding hypoglycemia [46].

In the case of patients who use CGM, the objective should be to reach time in range (70 to 180 mg/dL) of more than 70%, with time in hypoglycemia (<70 mg/dL) lower than 4%. The values for elderly or frail patients are reaching time in range >50% and time in hypoglycemia <1% [47,48].

#### 3.2.2. Glucose Monitoring

Control of glucose levels is essential if we are to maintain good glycemic control during infection and detect possible hyperglycemic complications or hypoglycemia. The number of checks depends on the type of DM, treatment, and degree of control. However, in the case of COVID-19 infection, the number of daily checks should be increased and the results analyzed. The use of CGM or FGM systems as well as glucose monitors with the option to download data and a cloud connection enables remote monitoring by health professionals.

In the case of CGM, it is important to remember the possible interference of paracetamol with some systems (Dexcom G5, Guardian Connect, Enlite-Guardian Link, Enlite-Guardian 2 Link, Guardian Sensor 3-Guardian Link 3, Eversense, etc.) [49]. In these cases, capillary blood glucose monitoring should be performed before taking paracetamol.

#### 3.2.3. Pharmacologic Treatment

Various glucose-lowering agents (e.g., metformin, DPP4 inhibitors, and GLP1 receptor agonists) have anti-inflammatory action, thus supporting the hypothesis that one or more of these drugs could be particularly useful in persons with T2D and COVID-19 [50]. However, in the absence of prospective randomized controlled trials, there continues to be insufficient evidence for stating whether the use of a specific class of glucose-lowering agents is beneficial or harmful for people with COVID-19 [51]. An analysis of COVID-19 results from 1317 persons with DM (88.5% with T2D) in French hospitals revealed no clear association between glucose-lowering agents and symptom severity [52]. Similarly, no significant association was found between treatment and clinical findings for COVID-19 infection in 1762 persons with T2D in the SEMI-COVID-19 registry in Spain [53].

Patients with mild COVID-19 can generally continue with their usual treatment, providing they maintain adequate oral tolerance with good fluid intake, and there are no contraindications for treatment. Nevertheless, factors such as kidney function, nutritional status, risk of hypoglycemia, severity of infection, and glycemic control itself may require treatment to be adjusted. The main recommendations for antihyperglycemic agents in patients with COVID-19 are summarized in Table 2 [25,37,48,50].

#### 3.2.4. Control of Other Cardiovascular Risk Factors

Angiotensin-converting enzyme inhibitors (ACEI) and angiotensin II receptor blockers (ARA2) are essential for management of hypertension, heart failure, and diabetic nephropathy. No clear evidence in favor of or against these agents in persons with T2D at risk of or infected by SARS-CoV-2 has been published to date, despite speculation over possible adverse effects [7]. There are clear risks associated with discontinuation since control of hypertension and protection against kidney disease may be compromised. At present, most international organizations recommend continuation of ACEI/ARA2 unless there are explicit contraindications, such as hypotension or acute kidney injury [58,59].

As for dyslipidemia, there is currently insufficient evidence in favor of or against continuation of statins in patients with DM and COVID-19. Increased liver and muscle enzymes have been associated with the infection [60], and some authors recommend monitoring creatine kinase in affected patients [61]. Decisions should be tailored taking into account the indication for statins as well as possible interactions with antiviral agents.

Risk of thrombosis should also be taken into account. Persons with DM are more likely to experience thrombosis, which is a relatively frequent complication in COVID-19 [60]. Treatment should be continued in patients taking antithrombotic agents. Similarly, in the absence of contraindications, all patients hospitalized with COVID-19 should receive prophylaxis for venous thromboembolism [62].

#### 3.2.5. Special Considerations in T1D

Persons with T1D should never discontinue insulin owing to the high risk of hyperosmolar hyperglycemic syndrome and diabetic ketoacidosis after infection [63]. It is essential to guarantee appropriate fluid intake and frequent monitoring of glucose levels and ketone bodies. Patients should be trained to know when to monitor ketones and be aware of the need for additional doses of insulin. In the case of blood ketone levels higher than 3 mmol/L, patients should consult their health professionals.

### 3.3. Hospitalized Patients with Diabetes and COVID-19

The COVID-19 pandemic has generated new challenges in hospital management of DM. Good control of glycemia helps to improve clinical outcomes although it also requires frequent contact between health care personnel and patients to ensure appropriate monitoring of blood glucose, administration of insulin, and resolution of hypoglycemia in a situation where it is recommended to minimize interactions with patients in order to avoid exposure to COVID-19 [64]. Table 3 summarizes the main recommendations for management of hyperglycemia in critically ill and non-critically ill patients depending on their clinical status.

#### 3.3.1. Management of Hyperglycemia in Critically Ill Patients with COVID-19

Glucose levels should be maintained between 140 and 180 mg/dL in most critically ill patients [66]; more rigorous targets (110–140 mg/dL) could be reasonable for selected patients, providing they can be reached without significant hypoglycemia [67,68,69,70].

Insulin should be the treatment of choice for critically ill patients with COVID-19 [71]. The most effective way of reaching glucose targets is continuous intravenous infusion based on validated written or computerized protocols [72,73]. Most protocols require glucose to be monitored at least once hourly, thus necessitating contact with staff. Exposure of health care staff managing hyperglycemia in critically ill patients with COVID-19 should be minimized. In the case of hemodynamically stable patients not receiving parenteral nutrition or high doses of corticosteroids, we recommend using subcutaneous insulin regimens (basal-bolus correction or basal correction) instead of intravenous regimens and monitoring blood glucose four times daily, together with other nursing care, in order to reduce the need to enter the patient’s room [65].

Transferring administration of insulin from intravenous to subcutaneous is recommended when the patient is clinically stable. The initial dose of subcutaneous insulin when switching can be calculated as 60–80% of the intravenous dose administered during the previous 24 h. Short-acting insulin can be administered for 1–2 h and long-acting insulin for 2–3 h before interrupting administration of intravenous insulin [74,75].

#### 3.3.2. Management of Hyperglycemia in Non-Critically Ill Patients with COVID-19

Glucose values before meals and after fasting <140 mg/dL with random maximum glucose <180 mg/dL could be appropriate in stable patients with mild disease and strict previous glycemic control, whereas glucose levels >180 mg/dL may be acceptable in patients with a high risk of hypoglycemia or limited life expectancy as a way of minimizing the risk of hypoglycemia [65].

Insulin is still considered the most appropriate drug for effective control of glycemia in hospital. A regimen with basal, prandial, and correctional components is the preferred approach in non-critically ill hospitalized patients with COVID-19 and good nutritional intake; basal insulin or basal insulin with correction doses is the best choice for patients whose oral intake cannot be guaranteed. Prolonged use of sliding-scale rapid-acting insulin as the only treatment for hyperglycemia is not recommended.

DPP4 inhibitors combined with basal insulin can be an alternative in patients with COVID-19 and mild-to-moderate hyperglycemia. The DARE-19 study recently showed that in patients with cardiometabolic risk factors hospitalized with COVID-19, treatment with dapagliflozin did not result in a statistically significant reduction in the risk of organ dysfunction or death. Similarly, it did not result in a significant improvement in clinical recovery although it was well tolerated. Therefore, these findings could support continuation of SGLT2i for patients already receiving them before a COVID-19 diagnosis as long as they are monitored [76]. 

Treatment of corticosteroid-induced hyperglycemia with insulin corticosteroids can aggravate or induce hyperglycemia in hospitalized patients with COVID-19 with and without DM [65,77]. As for management, some authors have reported their experience adding neutral protamine Hagedorn insulin at doses of 20–30 IU in the morning as well as the usual insulin regimen [71]. In our experience, the best option is to add the calculated increase in the dose of insulin, taking into account body weight, corticosteroid dose, and the patient’s usual total dose, which should be distributed according to the insulin regimen and the usual corticosteroid schedule [26].

#### 3.3.3. Glucose Monitoring of Patients with COVID-19 in Hospital

Control of DM in hospital usually requires multiple daily glucose readings. This is challenging for patients who are in isolation. Therefore, the United States Food and Drug Administration (FDA) has authorized self-monitoring of glucose in hospital by patients using their own glucose monitors during the COVID-19 pandemic [78] and is in favor of using CGM in non-critically ill patients [79,80,81] although this does not imply approval for use in hospital. Dexcom G6 and FreeStyle Libre have proven to reduce the incidence of hypoglycemia in non-critically ill patients [82,83,84]. Neither requires calibration of capillary glycemia, thus minimizing staff exposure and workload. Similarly, the fact that these devices are not affected by interference with paracetamol is yet another advantage in patients with COVID-19. A pilot study found that Dexcom G6 is feasible in non-critically ill COVID-19 patients, with a MARD of 9.77% [85]. Similar results have been described in critically ill hospitalized patients with COVID-19 [86].

Therefore, CGM and FGM could be considered for non-critically ill hospitalized patients with COVID-19 in order to limit the number of capillary glycemia tests, minimize staff exposure, and optimize glycemic control [64]. Potential candidates include patients with moderate-severe hyperglycemia requiring treatment with multiple doses of insulin, patients with high glycemic variability or risk of hypoglycemia, and patients with hyperglycemia that is difficult to manage, such as corticosteroid-induced hyperglycemia or hyperglycemia induced by artificial nutrition [87]. Similarly, persons using CGM or FGM as outpatients could continue to use their devices in hospital, providing protocols are in place, and there are staff trained in their management [88].

## 4. Care of Patients with Diabetes during the COVID-19 Pandemic and Afterwards

The pandemic led to unprecedented changes in clinical practice, including the closure of some primary care centers and restructuring of hospitals, with considerable resources aimed at management of patients with COVID-19 and a rapid transition to online care for other conditions. As the government was promoting measures to curb and contain the spread of the disease, health professionals faced the difficult task of managing risks both for patients and for themselves while learning to implement new remote care systems. In this setting, the first challenge was to maintain remote care, mainly by telephone, in order to address urgent situations and patients whose care could not be delayed as well as to adapt management protocols for hospitalized patients with DM to the special circumstances affecting hospitalization of patients with COVID-19. The second challenge was to plan health care after the initial phase in order not to postpone scheduled care and resume previously postponed activity via a face-to-face or remote visit.

New management protocols have been suggested for hospitalized patients with DM or hyperglycemia although information on the safety and efficacy of these protocols and their application is lacking [65]. While waiting for these strategies to be evaluated, and faced with the urgent need to implement effective approaches to glycemic control in hospitalized patients with COVID-19, we recently proposed a series of recommendations on management of hyperglycemia in the critical setting and noncritical care setting, taking into account factors such as the need to prevent staff exposure and the fact that many health professionals caring for patients with COVID-19 may be relatively unfamiliar with management of hyperglycemia [65].

With the sudden outbreak of the COVID-19 pandemic, those working in outpatient care have seen how the struggle against SARS-CoV-2 became a priority for the health system and how care of persons with chronic diseases, such as DM, was interrupted partially or completely. Health professionals responded with rapid and urgent adoption of alternative means of caring for patients, as follows: online visits via video calls and, more commonly, by telephone; easier access to prescription medication via online prescriptions; promotion of structured educational resources online; and the increase in the use of telemedicine tools that make it possible to transfer the results of glucose monitoring to health professionals (limited until relatively recently to patients with T1D). However, many patients had their visits and appointments for analyses and additional testing cancelled. In a survey on the impact of COVID-19 on persons with DM carried out by the Spanish Diabetes Federation (Federación Española de Diabetes (FEDE)) and the Spanish Diabetes Society (Sociedad Española de Diabetes (SED)), 46% of the 335 patients surveyed (59% with T1D) had their visit cancelled, and 40% had an online visit; furthermore, 78% felt that they would experience difficulties making changes to their treatment. An audit of more than 125.8 million primary care visits from the US National Disease and Therapeutic Index showed that visit frequency decreased by 21.4% during the second quarter of 2020 compared with the mean quarterly visit volume for the second quarters of 2018 and 2019, which were not compensated for by remote visits. In addition, evaluation of risk factors was less common during the remote visits than during face-to-face visits [89]. Similarly, changes in care were implemented without clear guidelines or planning and mainly involved glycemia but not other comorbid conditions, such as obesity, hypertension, dyslipidemia, and cardiovascular disease. Moreover, evidence on the efficacy of these strategies in the case of the COVID-19 pandemic is scarce. A meta-analysis of randomized controlled clinical trials that compared telemedicine-based interventions with standard care found that systems enabling adjustments to medication with or without text messages or a web page improved glycosylated hemoglobin but not other clinically relevant outcomes in patients with DM [90]. The most recent meta-analysis, which included eight studies based on remote visits and 34 based on remote monitoring in patients with T1D and T2D, revealed that telemedicine-based interventions were more effective than standard care for control of DM [91].

One positive aspect is that the disruptive effect of the pandemic has led to the rapid disappearance of barriers to the use of remote care tools, thus making telemedicine another option in the care of chronically ill patients in the future. The pandemic has considerably promoted the use of already available but rarely used applications and platforms, which enable patients to upload data from their glucose monitors, CGM devices, or insulin pumps so that their doctors can make decisions on treatment. In addition, health professionals and probably health care organizations have recognized the value of some of these profound changes in a sector such as that of health care, where habits are hard to change. However, there are major limitations to telemedicine becoming a powerful tool in the care of patients with DM. First, efficient use of this approach is currently restricted to patients who are fully able to manage the necessary technology and are seen at centers whose professionals are skilled in the use of this technology. In most cases, remote care is limited to telephone calls. The FEDE/SED survey showed that 41% of patients considered remote visits to be poorly efficient or inefficient, and 57% preferred their future health care to be based on a mix of face-to-face and remote visits. Similarly, many of the tools were used on an improvised basis, without previous cost-effectiveness studies and without changes in the care model to make efficient application easier. Research on telemedicine during the pandemic, while generating some knowledge, has generally been based on small or nonrepresentative samples and limited to analysis of frequency of use although with few data on content and safety and efficacy.

The question many of us ask today is whether this greater use of remote care will be maintained once the health care crisis brought about by COVID-19 has passed. The answer is not easy to predict although it seems foolish not to take advantage of what we have learned from the rapid deployment of telemedicine, especially if we consider that this is a broad area with room for development. Furthermore, while not replacing face-to-face visits, telemedicine can facilitate processes, streamline the system, and provide valuable information for health professionals and their patients. Nevertheless, it is also clear that for telemedicine to become a reality, more widespread adoption as a result of the pandemic is not sufficient, and a series of initiatives will be needed to improve it, as follows:

- Development and broad implementation of remote diagnostic and treatment technologies that make it possible to obtain variables of interest for management of DM from the patient in his/her usual environment;
- General electronic clinical histories in health services that include all the information generated during the patient’s lifetime. System standardization and interoperability are essential if we are to ensure real integration and coordination between various care levels;
- Establishment of universal mechanisms and protocols for transferring data on variables of interest independently of where they are generated to the clinical history in standard format and in such a way that they are easily interpretable. The complexity and poor user-friendliness of the approach use up health professionals’ time and are among the main reasons this modality is discontinued. All of these actions are essential so that health professionals spend more time making decisions and less time recording data, thus leading to greater clinical efficacy and reduced resistance to change by professionals;
- In parallel, all those involved should be trained to avoid the digital gap between patients and professionals, and collaboration between professionals and care levels should be encouraged by providing reimbursement for the use of digital technology and models for evaluation and assignment of resources that discourage silo working;
- As for any other medical intervention, it is necessary to generate evidence on use—in terms of cost-effectiveness and implementation challenges—of new telemedicine systems and strategies by comparing them with previously used approaches.

If these aspects are not taken into account, we will probably miss the opportunity brought about by the COVID-19 pandemic to achieve the real goal, that is, to transform our health care model so that it can respond to the challenges the system faces in providing health care to persons with chronic diseases, such as DM.

While the long-term implications of COVID-19 in persons with DM are unknown, available data indicate that even short-term interruption of care could prove catastrophic. The impact is especially important in older persons from disadvantaged areas with reduced ability to self-monitor and self-adjust treatment [30]. Prolongation of the pandemic and restrictions in effective clinical care will worsen the situation. In order to minimize the consequences of this situation, it is necessary to guarantee that patients receive efficient clinical care that takes into account various services, including screening for the disease in at-risk persons, education, and monitoring of control and complications at face-to-face or remote visits as well as adaptation of treatment of DM in the setting of COVID-19.

## 5. Conclusions

Diabetes is one of the most common comorbidities linked to COVID-19, and there is consistent evidence that diabetes increases the risk of severe COVID-19 disease, including admission to the intensive care unit and death. Socioeconomic disadvantage and higher blood glucose levels are associated with worse COVID-19 outcomes. Tight control of glucose levels could prove crucial in patients with diabetes mellitus to prevent progression to severe COVID-19. However, data on the effect of insulin and non-insulin anti-diabetic drugs are lacking. To minimize the consequences of this situation, it is crucial to guarantee that patients remain engaged with diabetes services and receive efficient clinical care, including screening for the disease, education, and monitoring of control and complications at face-to-face or remote visits. Treatment of diabetes must be adapted appropriately to the COVID-19 setting. 

This article summarizes recommendations for management of diabetes in various situations during the COVID-19 pandemic and could serve as a guide for healthcare providers to ensure continuity of care for people with diabetes. More research is needed on the acute and long-term effects of COVID-19 in this population. It is also necessary to assess the impact of these recommendations and the implementation of technological advances in the deployment of telemedicine and management of diabetes in inpatient and outpatient care. 

## Figures and Tables

**Table 1:** General recommendations for the prevention of COVID-19 in persons with diabetes [37].

**Hygiene and social Distancing Measures**
**Lifestyle**
Healthy diet: limit refined sugar and fat; avoid snacks [40,41] Physical exercise: avoid a sedentary lifestyle; take regular aerobic exercise (walking, cycling, etc.) combined with strength exercise (weights, resistance bands, pushing exercises, etc.) [42,43]. Avoid smoking; avoid alcohol Stress management
**Glycemic control**
Regular monitoring of blood glucose levels Continue with regular treatment, except in the case of contraindications Consider dose adjustment (insulin, sulfonylureas, etc.) depending on diet and physical activity Ketone level monitoring in T1D, especially if hyperglycemia is persistent (>250 mg/dL), or there are symptoms suggestive of ketoacidosis (nausea, vomiting, abdominal pain, etc.)
**Control of comorbid conditions** (obesity, blood pressure, dyslipidemia, etc.)
**Vaccination** (routine vaccination against pneumococcal and seasonal influenza)
**Minimize exposure to SARS-CoV-2** (prioritize telemedicine)

SARS-CoV-2, severe acute respiratory syndrome coronavirus 2.

**Table 2:** Recommendations on the use of antihyperglycemic drugs in patients with diabetes and COVID-19 [37,48,54,55,56,57].

Treatment	Clinical Recommendation	Special Considerations in COVID-19
**Metformin**	Suspend in severe cases, with hemodynamic instability or hypoxia	Risk of lactic acidosis in hypoxia and acute disease Monitor kidney function. Suspend if glomerular filtration <30 mL/min/1.73 m^2^
**Sulfonylureas**	Suspend in cases where food intake is not guaranteed owing to the risk of hypoglycemia	The risk of hypoglycemia may be greater with concomitant use of treatments such as hydroxychloroquine
**DPP4i**	Continue in outpatients Potential option in hospitalized patients with mild hyperglycemia, combined with basal insulin	Favorable safety profile and possible use in kidney failure
**GLP1ra**	Suspend in severe cases	Risk of dehydration in the case of severe gastrointestinal adverse effects (nausea, vomiting, etc.) Maintain a regular diet and ensure good hydration
**SGLT2i**	Suspend in severe cases, or if food/fluid intake cannot be guaranteed	Risk of euglycemic diabetic ketoacidosis induced by dehydration and insulin deficiency Preserving cardiovascular and kidney function is critical for ensuring favorable progress of COVID-19 in persons with diabetes
**Glitazones**	Suspend in severe cases with hemodynamic instability or heart of liver dysfunction	Risk of fluid retention and heart failure
**Insulin**	Continue treatment of choice in hospitalized patients. Adjust dose depending on glycemic control, risk of hypoglycemia, severity of infection, and concomitant treatment Requires frequent monitoring of blood glucose (capillary glycemia or CGM/FGM)	Insulin requirements may be very high in hospitalized patients with severe infection

GLP1ra, glucagon-like peptide 1 receptor agonist; DPP4i, dipeptidyl peptidase 4 inhibitor; SGLT2i, sodium-glucose co- transporter-2 inhibitors; CGM, continuous glucose monitoring; FGM, flash glucose monitoring.

**Table 3:** Management of hyperglycemia in critically ill and non-critically ill patients with COVID-19 [65].

	Blood Glucose Target	Clinical Situation		Insulin Regimen	Glucose Monitoring
**Critically ill patients**	140–180 mg/dL *	Hemodynamically unstable Parenteral nutrition Varying insulin requirements Treatment with corticosteroids		Continuous intravenous insulin infusion	Every hour
		Hemodynamically stable Stable insulin requirements		Subcutaneous insulin Basal + correction or basal-bolus + correction	Every 4–6 h
**Non-critically ill patients**	110–180 mg/dL **	T1D T2D with OAD ± insulin	No oral intake	Basal insulin + correction	Every 4–6 h ****
			Oral intake	Basal-bolus insulin + correction	Before meals and at bedtime ****
		T2D with diet DM not known	Glycemia on admission <180 mg/dL	Correction insulin dose before meals or every 6 h ***	Before meals and at bedtime or every 6 h ****
			Glycemia on admission >180 mg/dL	Basal-bolus insulin + correction	Before meals and at bedtime ****

* 110–140 mg/dL may be reasonable for selected patients, providing it can be reached without hypoglycemia. ** 110–140 mg/dL may be reasonable in patients with mild disease and good previous glycemic control. Blood glucose >180 mg/dL may be reasonable for patients with high risk of hypoglycemia or limited life expectancy. *** In order to calculate insulin requirements during the first 24 h. Afterwards, intensify to basal correction or basal- bolus correction regimen. **** Consider using continuous glucose monitoring, if possible, in order to limit the number of capillary blood glucose controls. OAD, oral antidiabetic agents; DM, diabetes mellitus; T1D, diabetes mellitus type 1; T2D, diabetes mellitus type 2.

## References

[1] Fadini G.P., Morieri M.L., Longato E., Avogaro A. (2020). Prevalence and impact of diabetes among people infected with SARS- CoV-2. J. Endocrinol. Investig..

[2] Roncon L., Zuin M., Rigatelli G., Zuliani G. (2020). Diabetic patients with COVID-19 infection are at higher risk of ICU admission and poor short-term outcome. J. Clin. Virol..

[3] Singh A.K., Gupta R., Ghosh A., Misra A. (2020). Diabetes in COVID-19: Prevalence, pathophysiology, prognosis and practical considerations. Diabetes Metab. Syndr..

[4] Li B., Yang J., Zhao F., Zhi L., Wang X., Liu L., Bi Z., Zhao Y. (2020). Prevalence and impact of cardiovascular metabolic diseases on COVID-19 in China. Clin. Res. Cardiol..

[5] Barron E., Bakhai C., Kar P., Weaver A., Bradley D., Ismail H., Knighton P., Holman N., Khunti K., Sattar N. (2020). Associations of type 1 and type 2 diabetes with COVID-19-related mortality in England: A whole-population study. Lancet Diabetes Endocrinol..

[6] Zhou F., Yu T., Du R., Fan G., Liu Y., Liu Z., Xiang J., Wang Y., Song B., Gu X. (2020). Clinical course and risk factors for mortality of adult inpatients with COVID-19 in Wuhan, China: A retrospective cohort study. Lancet.

[7] Fang L., Karakiulakis G., Roth M. (2020). Are patients with hypertension and diabetes mellitus at increased risk for COVID-19 infection?. Lancet Respir. Med..

[8] Moazzami B., Chaichian S., Kasaeian A., Djalalinia S., Akhlaghdoust M., Eslami M., Broumand B. (2020). Metabolic risk factors and risk of COVID-19: A systematic review and meta-analysis. PLoS ONE.

[9] Huang I., Lim M.A., Pranata R. (2020). Diabetes mellitus is associated with increased mortality and severity of disease in COVID-19 pneumonia. A systematic review, meta-analysis, and meta-regression. Diabetes Metab. Syndr..

[10] Bloomgarden Z.T. (2020). Diabetes and COVID-19. J. Diabetes.

[11] Bartsch S.M., Ferguson M.C., McKinnell J.A., O’Shea K.J., Wedlock P.T., Siegmund S.S., Lee B.Y. (2020). The potential health care costs and resource use associated with COVID-19 in The United States. Health Aff..

[12] Muniyappa R., Gubbi S. (2020). Covid-19 pandemic, coronaviruses, and diabetes mellitus. Am. J. Physiol. Endocrinol. Metab..

[13] Klonoff D.C., Messler J.C., Umpiérrez G.E., Peng L., Booth R., Crowe J., Garrett V., McFarland R., Pasquel F.J. (2021). Association between achieving inpatient glycemic control and clinical outcomes in hospitalized patients with COVID-19: A multicenter, retrospective hospital-based analysis. Diabetes Care.

[14] Carrasco-Sánchez F.J., López-Carmona M.D., Martínez-Marcos F.J., Pérez-Belmonte L.M., Hidalgo-Jiménez A., Buonaiuto V., Fernández Suárez C., Freire Castro S.J., Luordo D., Pesqueira Fontan P.M. (2021). Admission hyperglycaemia as a predictor of mortality in patients hospitalized with COVID-19 regardless of diabetes status: Data from the Spanish SEMI-COVID-19 Registry. Ann. Med..

[15] Sardu C., D’Onofrio N., Balestrieri M.L., Barbieri M., Rizzo M.R., Messina V., Maggi P., Coppola N., Paolisso G., Marfella R. (2020). Outcomes in patients with hyperglycemia affected by COVID-19: Can we do more on glycemic control?. Diabetes Care.

[16] Iacobellis G. (2020). COVID-19 and diabetes: Can DPP4 inhibition play a role?. Diabetes Res. Clin. Pract..

[17] Rao S., Lau A., Hon-Cheong S. (2020). Exploring diseases/traits and blood proteins causally related to expression of ACE2, the putative receptor of SARS-CoV-2: A Mendelian randomization analysis highlights tentative relevance of diabetes-related traits. Diabetes Care.

[18] Bindom S.M., Lazartigues E. (2009). The sweeter side of ACE2: Physiological evidence for a role in diabetics. Mol. Cell Endocrinol..

[19] Bode B., Garrett V., Messler J., McFarland R., Crowe J., Booth R., Klonoff D.C. (2020). Glycemic characteristics and clinical outcomes of COVID-19 patients hospitalized in the United States. J. Diabetes Sci. Technol..

[20] Zhou J., Tan J. (2020). Diabetes patients with COVID-19 need better blood glucose management in Wuhan, China. Metabolism.

[21] Wu L., Girgis C.M., Cheung N.W. (2020). COVID-19 and diabetes: Insulin requirements parallel illness severity in critically unwell patients. Clin. Endocrinol..

[22] Pal R., Banerjee M., Yadav U., Bhattacharjee S. (2020). Clinical profile and outcomes in COVID-19 patients with diabetic ketoacidosis: A systematic review of literature. Diabetes Metab. Syndr..

[23] Yang J.K., Lin S.S., Ji X.J., Guo L.M. (2010). Binding of SARS coronavirus to its receptor damages islets and causes acute diabetes. Acta Diabetol..

[24] Li J., Wang X., Chen J., Zuo X., Zhang H., Deng A. (2020). COVID-19 infection may cause ketosis and ketoacidosis. Diabetes Obes. Metab..

[25] Lim S., Bae J.H., Kwon H.S., Nauck M.A. (2021). COVID-19 and diabetes mellitus: From pathophysiology to clinical management. Nat. Rev. Endocrinol..

[26] Perez A., Jansen-Chaparro S., Saigi I., Bernal-López M.R., Miñambres I., Gómez-Huelgas R. (2014). Glucocorticoid-induced hyperglycemia. J. Diabetes.

[27] Scheen A.J., Marre M., Thivolet C. (2020). Prognostic factors in patients with diabetes hospitalized for COVID-19: Findings from the CORONADO study and other recent reports. Diabetes Metab..

[28] Fernández E., Cortázar A., Bellido V. (2020). Impact of COVID-19 lockdown on glycemic control in patients with type 1 diabetes. Diabetes Res. Clin. Pract..

[29] Capaldo B., Annuzzi G., Creanza A., Giglio C., De Angelis R., Lupoli R., Masulli M., Riccardi G., Albarosa Rivellese A., Bozzetto L. (2020). Blood glucose control during lockdown for COVID-19: CGM metrics in Italian adults with type 1 diabetes. Diabetes Care.

[30] Garofolo M., Aragona M., Rodia C., Falcetta P., Bertolotto A., Campi F., Del Prato S., Penno G. (2021). Glycaemic control during the lockdown for COVID-19 in adults with type 1 diabetes: A meta-analysis of observational studies. Diabetes Res. Clin. Pract..

[31] Fisher L., Polonsky W., Asuni A., Jolly Y., Hessler D. (2020). The early impact of the COVID-19 pandemic on adults with type 1 or type 2 diabetes: A national cohort study. J. Diabetes Complicat..

[32] Tejera-Pérez C., Moreno-Pérez Ó., Ríos J., Reyes-García R. (2021). People living with type 1 diabetes point of view in COVID-19 times (covidT1 study): Disease impact, health system pitfalls and lessons for the future. Diabetes Res. Clin. Pract..

[33] Biancalana E., Parolini F., Mengozzi A., Solini A. (2021). Short-term impact of COVID-19 lockdown on metabolic control of patients with well-controlled type 2 diabetes: A single-centre observational study. Acta Diabetol..

[34] Carr M.J., Wright A.K., Leelarathna L., Thabit H., Milne N., Kanumilli N., Ashcroft D.M., Rutter M.K. (2021). Impact of COVID-19 on diagnoses, monitoring and mortality in people with type 2 diabetes: A UK-wide cohort study involving 14 million people in primary care. Lancet Diabetes Endocrinol..

[35] Jacob L., Rickwood S., Rathmann W., Kostev K. (2020). Change in glucose-lowering medication regimens in individuals with type 2 diabetes mellitus during the COVID-19 pandemic in Germany. Diabetes Obes. Metab..

[36] Wang A., Zhao W., Xu Z., Gu J. (2020). Timely blood glucose management for the outbreak of 2019 novel coronavirus disease (COVID-19) is urgently needed. Diabetes Res. Clin. Pract..

[37] Katulanda P., Dissanayake H.A., Ranathunga I., Ratnasamy V., Wijewickrama P.S.A., Yogendranathan N., Gamage K.K.K., de Silva N.L., Sumanatilleke M., Somasundaram N. (2020). Prevention and management of COVID-19 among patients with diabetes: An appraisal of the literature. Diabetologia.

[38] Pal R., Bhadada S.K., Misra A. (2021). COVID-19 vaccination in patients with diabetes mellitus: Current concepts, uncertainties and challenges. Diabetes Metab. Syndr..

[39] Marfella R., D’Onofrio N., Sardu C., Scisciola L., Maggi P., Coppola N., Romano C., Messina V., Turriziani F., Siniscalchi M. (2021). Does poor glycaemic control affect the immunogenicity of the COVID-19 vaccination in patients with type 2 diabetes: The CAVEAT study. Diabetes Obes. Metab..

[40] Pascual Fuster V., Pérez Pérez A., Carretero Gómez J., Caixàs Pedragós A., Gómez-Huelgas R., Pérez-Martínez P. (2021). Executive summary: Updates to the dietary treatment of prediabetes and type 2 diabetes mellitus. Clin. Investig. Arteriosler..

[41] Reyes-García R., Moreno-Pérez Ó., Tejera-Pérez C., Fernández-García D., Bellido-Castañeda V., De la Torre Casares M.L., Rozas-Moreno P., Fernández-García J.C., Martínez Marco A., Escalada-San Martín J. (2019). Documento de abordaje integral de la diabetes tipo 2. Endocrinol. Diabetes Nutr..

[42] Marçal I.R., Fernandes B., Viana A.A., Ciolac E.G. (2020). The urgent need for recommending physical activity for the management of diabetes during and beyond COVID-19 outbreak. Front. Endocrinol..

[43] Philippou A., Chryssanthopoulos C., Maridaki M., Dimitriadis G., Koutsilieris M., Kokkinos P., Narayan P. (2010). Exercise metabolism in health and disease. Cardiorespiratory Fitness in Cardiometabolic Diseases.

[44] Koliaki C., Tentolouris A., Eleftheriadou I., Melidonis A., Dimitriadis G., Tentolouris N. (2020). Clinical management of diabetes mellitus in the era of COVID-19: Practical issues, peculiarities and concerns. J. Clin. Med..

[45] Hussain A., Bhowmik B., Do Vale Moreira N.C. (2020). COVID-19 and diabetes: Knowledge in progress. Diabetes Res. Clin. Pract..

[46] American Diabetes Association (2021). 6. Glycemic targets-Standards of medical care in diabetes—2021. Diabetes Care.

[47] Battelino T., Danne T., Bergenstal R.M., Amiel S.A., Beck R., Biester T., Bosi E., Buckingham B.A., Cefalu W.T., Close K.L. (2019). Clinical targets for continuous glucose monitoring data interpretation: Recommendations from the international consensus on time in range. Diabetes Care.

[48] Bornstein S.R., Rubino F., Khunti K., Mingrone G., Hopkins D., Birkenfeld A.L., Boehm B., Amiel S., Holt R.I., Skyler J.S. (2020). Practical recommendations for the management of diabetes in patients with COVID-19. Lancet Diabetes Endocrinol..

[49] Maahs D.M., DeSalvo D., Pyle L., Ly T., Messer L., Clinton P., Westfall E., Wadwa R.P., Buckingham B. (2015). Effect of acetaminophen on CGM glucose in an outpatient setting. Diabetes Care.

[50] Sun B., Huang S., Zhou J. (2021). Perspectives of antidiabetic drugs in diabetes with coronavirus infections. Front. Pharmacol..

[51] Finan B., Yang B., Ottaway N., Smiley D.L., Ma T., Clemmensen C., Chabenne J., Zhang L., Habegger K.M., Fischer K. (2015). A rationally designed monomeric peptide triagonist corrects obesity and diabetes in rodents. Nat. Med..

[52] Cariou B., Hadjadj S., Wargny M., Pichelin M., Al-Salameh A., Allix I., Amadou C., Arnault G., Baudoux F., Bauduceau B. (2020). Phenotypic characteristics and prognosis of inpatients with COVID-19 and diabetes: The CORONADO study. Diabetologia.

[53] Pérez-Belmonte L.M., Torres-Peña J.D., López-Carmona M.D., Ayala-Gutiérrez M.M., Fuentes-Jiménez F., Huerta L.J., Muñoz J.A., Rubio-Rivas M., Madrazo M., Guzmán García M. (2020). Mortality and other adverse outcomes in patients with type 2 diabetes mellitus admitted for COVID-19 in association with glucose-lowering drugs: A nationwide cohort study. BMC Med..

[54] DeFronzo R., Fleming G.A., Chen K., Bicsak T.A. (2016). Metformin-associated lactic acidosis: Current perspectives on causes and risk. Metabolism.

[55] Vitale R.J., Valtis Y.K., McDonnell M.E., Palermo N.E., Fisher N.D.L. (2021). Euglycemic diabetic ketoacidosis with COVID-19 infection in patients with type 2 diabetes taking SGLT2 inhibitors. AACE Clin. Case Rep..

[56] Schwartz S., DeFronzo R.A. (2013). Is incretin-based therapy ready for the care of hospitalized patients with type 2 diabetes? The time has come for GLP-1 receptor agonists!. Diabetes Care.

[57] Longo M., Caruso P., Maiorino M.I., Bellastella G., Giugliano D., Esposito K. (2020). Treating type 2 diabetes in COVID-19 patients: The potential benefits of injective therapies. Cardiovasc. Diabetol..

[58] Vaduganathan M., Vardeny O., Michel T., McMurray J.J.V., Pfeffer M.A., Solomon S.D. (2020). Renin–angiotensin–aldosterone system inhibitors in patients with Covid-19. N. Engl. J. Med..

[59] Patel A.B., Verma A. (2020). COVID-19 and angiotensin-converting enzyme inhibitors and angiotensin receptor blockers: What is the evidence?. JAMA.

[60] Bangash M.N., Patel J., Parekh D. (2020). COVID-19 and the liver: Little cause for concern. Lancet Gastroenterol. Hepatol..

[61] Ceriello A., Standl E., Catrinoiu D., Itzhak B., Lalic N.M., Rahelic D., Schnell O., Škrha J., Valensi P. (2020). Diabetes and Cardiovascular Disease (D&CVD) EASD Study Group. Issues of cardiovascular risk management in people with diabetes in the COVID-19 era. Diabetes Care.

[62] Rahimi L., Malek M., Ismail-Beigi F., Khamseh M.E. (2020). Challenging issues in the management of cardiovascular risk factors in diabetes during the COVID-19 pandemic: A review of current literature. Adv. Ther..

[63] Ebekozien O.A., Noor N., Gallagher M.P., Alonso G.T. (2020). Type 1 diabetes and COVID-19: Preliminary findings from a multicenter surveillance study in the U.S. Diabetes Care.

[64] Korytkowski M., Antinori-Lent K., Drincic A., Hirsch I.B., McDonnell M.E., Rushakoff R., Muniyappa R. (2020). A pragmatic approach to inpatient diabetes management during the COVID-19 pandemic. J. Clin. Endocrinol. Metab..

[65] Bellido V., Pérez A. (2021). Inpatient hyperglycemia management and COVID-19. Diabetes Ther..

[66] American Diabetes Association (2020). 15 Diabetes care in the hospital-Standards of medical care in diabetes—2020. Diabetes Care.

[67] Umpiérrez G., Cardona S., Pasquel F., Jacobs S., Peng L., Unigwe M., Newton C.A., Smiley-Byrd D., Vellanki P., Halkos M. (2015). Randomized controlled trial of intensive versus conservative glucose control in patients undergoing coronary artery bypass graft surgery: GLUCO-CABG Trial. Diabetes Care.

[68] Krinsley J.S., Preiser J.C., Hirsch I.B. (2017). Safety and efficacy of personalized glycemic control in critically ill patients: A 2-year before and after intervention trial. Endocr. Pract..

[69] Pérez A., Ramos A., Carreras G. (2020). Insulin therapy in hospitalized patients. Am. J. Ther..

[70] Pasquel F.J., Umpiérrez G.E. (2020). Individualizing inpatient diabetes management during the coronavirus disease 2019 pandemic. J. Diabetes Sci. Technol..

[71] Hamdy O., Gabbay R.A. (2020). Early observation and mitigation of challenges in diabetes management of COVID-19 patients in critical care units. Diabetes Care.

[72] Moghissi E.S., Korytkowski M.T., DiNardo M., Einhorn D., Hellman R., Hirsch I.B., Inzucchi S.E., Ismail-Beigi F., Kirkman M.S., Umpierrez G.E. (2009). American Association of Clinical Endocrinologists and American Diabetes Association consensus statement on inpatient glycemic control. Diabetes Care.

[73] Pérez Pérez A., Conthe Gutiérrez P., Aguilar Diosdado M., Bertomeu Martínez V., Galdos Anuncibay P., García de Casasola G., Gomis de Bárbara R., Palma Gamiz J.L., Puig Domingo M., Sánchez Rodríguez A. (2009). Hospital management of hyperglycemia. Med. Clin..

[74] Avanzini F., Marelli G., Donzelli W., Busi G., Carbone S., Bellato L., Colombo E.L., Foschi R., Riva E., Roncaglioni M.C. (2011). Transition from intravenous to subcutaneous insulin: Effectiveness and safety of a standardized protocol and predictors of outcome in patients with acute coronary syndrome. Diabetes Care..

[75] Ramos A., Zapata L., Vera P., Betbese A.J., Pérez A. (2017). Transition from intravenous insulin to subcutaneous long-acting insulin in critical care patients on enteral or parenteral nutrition. Endocrinol. Diabetes Nutr..

[76] Kosiborod M.N., Esterline R., Furtado R.H.M., Oscarsson J., Gasparyan S.B., Koch G.G., Martinez F., Mukhtar O., Verma S., Chopra V. (2021). Dapagliflozin in patients with cardiometabolic risk factors hospitalized with COVID-19 (DARE-19): A randomized, double-blind, placebo-controlled, phase 3 trial. Lancet Diabetes Endocrinol..

[77] Mehta P., McAuley D.F., Brown M., Sánchez E., Tattersall R.S., Manson J.J., HLH Across Specialty Collaboration, UK (2020). COVID-19: Consider cytokine storm syndromes and immunosuppression. Lancet.

[78] FDA (2020). FAQs on Home-Use Blood Glucose Meters Utilized within Hospitals during the COVID-19 Pandemic. https://www.fda.gov/medical-devices/coronavirus-covid-19-and-medical-devices/using-home-use-blood-glucose-meters-hospitals-during-covid-19-pandemic.

[79] Welsh J.B., Hu G., Walker T.C., Sharma N., Cherñavvsky D. (2020). Glucose monitoring and diabetes management in the time of coronavirus disease 2019. J. Diabetes Sci. Technol..

[80] Dexcom (2020). Fact Sheet for Healthcare Providers: Use of Dexcom Continuous Glucose Monitoring Systems during the COVID-19 Pandemic. https://www.dexcom.com/hospitalfacts.

[81] Abbott (2020). Press release. FreeStyle Libre: Diabetes Care during COVID-19. https://www.abbott.com/corpnewsroom/product-and-innovation/freestyle-libre-diabetes-care-during-covid-19.html.

[82] Galindo R.J., Migdal A.L., Davis G.M., Urrutia M.A., Albury B., Zambrano C., Vellanki P., Pasquel F.J., Fayfman M., Peng L. (2020). Comparison of the FreeStyle Libre pro flash continuous glucose monitoring (CGM) system and point-of-care capillary glucose testing in hospitalized patients with type 2 diabetes treated with basal-bolus insulin regimen. Diabetes Care.

[83] Fortmann A.L., Spierling Bagsic S.R., Talavera L., García I.M., Sandoval H., Hottinger A., Philis-Tsimikas A. (2020). Glucose as the fifth vital sign: A randomized controlled trial of continuous glucose monitoring in a non-ICU hospital setting. Diabetes Care.

[84] Singh L.G., Satyarengga M., Marcano I., Scott W.H., Pinault L.F., Feng Z., Sorkin J.D., Umpierrez G.E., Spanakis E.K. (2020). Reducing inpatient hypoglycemia in the general wards using real-time continuous glucose monitoring: The glucose telemetry system, a randomized clinical trial. Diabetes Care.

[85] Reutrakul S., Genco M., Salinas H., Sargis R.M., Paul C., Eisenberg Y., Fang J., Caskey R.N., Henkle S., Fatoorehchi S. (2020). Feasibility of inpatient continuous glucose monitoring during the COVID-19 pandemic: Early experience. Diabetes Care.

[86] Agarwal S., Mathew J., Davis G.M., Shephardson A., Levine A., Louard R., Urrutia A., Perez-Guzman C., Umpierrez G.E., Peng L. (2021). Continuous glucose monitoring in the intensive care unit during the COVID-19 pandemic. Diabetes Care.

[87] Galindo R.J., Aleppo G., Klonoff D.C., Spanakis E.K., Agarwal S., Vellanki P., Olson D.E., Umpierrez G.E., Davis G.M., Pasquel F.J. (2020). Implementation of continuous glucose monitoring in the hospital: Emergent considerations for remote glucose monitoring during the COVID-19 pandemic. J. Diabetes Sci. Technol..

[88] Galindo R.J., Umpiérrez G.E., Rushakoff R.J., Basu A., Lohnes S., Nichols J.H., Spanakis E.K., Espinoza J., Palermo N.E., Awadjie D.G. (2020). Continuous glucose monitors and automated insulin dosing systems in the Hospital Consensus Guideline. J. Diabetes Sci. Technol..

[89] Alexander G.C., Tajanlangit M., Heyward J., Mansour O., Qato D.M., Stafford R.S. (2020). Use and content of primary care office-based vs telemedicine care visits during the COVID-19 pandemic in the US. JAMA Netw. Open.

[90] Faruque L.I., Wiebe N., Ehteshami-Afshar A., Liu Y., Dianati-Maleki N., Hemmelgarn B.R., Manns B.J., Tonelli M., Alberta Kidney Disease Network (2017). Effect of telemedicine on glycated hemoglobin in diabetes: A systematic review and meta-analysis of randomized trials. CMAJ..

[91] Tchero H., Kangambega P., Briatte C., Brunet-Houdard S., Retali G.-R., Rusch E. (2019). Clinical effectiveness of telemedicine in diabetes mellitus: A meta-analysis of 42 randomized controlled trials. Telemed. J. E Health.

